# Service system and care pathway of forensic psychiatry patients-international research project 2023-2026

**DOI:** 10.1192/j.eurpsy.2024.346

**Published:** 2024-08-27

**Authors:** R. Askola, O. Louheranta, A. Seppänen, T. Lantta

**Affiliations:** ^1^Department of Nursing Science, University of Turku, Turku; ^2^Niuvanniemi Hospital, Kuopio, Finland

## Abstract

**Introduction:**

The Finnish forensic psychiatric service system lacks the standards and criteria guiding the quality and contents of patient care. Ensuring best recovery-oriented practices in forensic psychiatric services need to be developed at several levels.

**Objectives:**

The purpose of this research project is to develop safe, high-quality psychiatric care. The outcome of this project is the production of quality criteria for the forensic psychiatric care and service system.

**Methods:**

The study will be executed at the Department of Nursing Science of the University of Turku during 2023-2026. The research methods include a literature review, a survey based on validated measurement questionnaires (Downes Survey, QPC-FIP, QPC-FIPS), individual and group interviews as well as the Delphi method. The research will cover the multidisciplinary employees at adult psychiatric wards in Finland’s larger hospital districts, employees of forensic psychiatric hospitals, and patients of forensic psychiatric hospitals. International specialists and specialists within Finland from various fields (nursing, medicine, psychology) will be invited to partake in the expert panel.

**Results:**

The research results will allow the development of the service system for forensic psychiatric patients in such a way that the identification of so-called risk patients can be improved already at the early stages of treatment, at the general psychiatric level. Moreover, the substance of care and participation during care can be created and the care following inpatient care and the patient’s transfer out of forensic psychiatric care can be developed. The research may promote the effectiveness of treatment by highlighting areas in the care chains that, when reinforced, will allow patients to receive the right kind of treatment at the right time. A proposal of standardized operating methods and quality criteria will be created for the Finnish forensic psychiatric treatment system. The research project will also reveal previously unresearched information that can be utilized in national health policy.

**Conclusions:**

The project will promote equal prospects for wellbeing and a participatory society for citizens by exploring the views of forensic psychiatric patients and thus developing forensic psychiatric services. The project will promote sustainable employment by exploring the views of psychiatric personnel and increasing the quality and safety of psychiatric services.

**Disclosure of Interest:**

None Declared

